# Review on epidemiology, disease burden, and treatment patterns of IgA nephropathy in select APAC countries

**DOI:** 10.1186/s12882-024-03555-5

**Published:** 2024-04-16

**Authors:** Omer Zaidi, Fen Du, Zhaoli Tang, Sandipan Bhattacharjee, Kristin Pareja

**Affiliations:** 1 OPEN Health, Boston, MA USA; 2 OPEN Health, Shanghai, China; 3 Otsuka Pharmaceutical Development & Commercialization, Inc., NJ Princeton, USA

**Keywords:** IgA nephropathy, Asia, Epidemiology, Treatment patterns, Disease burden, Review

## Abstract

**Background:**

Immunoglobulin type A (IgA) nephropathy is the most common primary glomerulonephritis (GN) worldwide with higher rates in East and Pacific Asia compared to North America and Europe. Despite high reported prevalence of IgAN in these countries, the overall disease prevalence across Asia is not available. Treatment patterns of IgAN patients across Asian countries have also not been summarized. The aim of this study was to review and summarize evidence on IgA nephropathy prevalence, treatment patterns, and humanistic and economic burden in mainland China, Taiwan, South Korea, Japan, and Australia.

**Methods:**

A targeted literature review was conducted in PubMed and local databases in China (including Taiwan), South Korea, Japan, and Australia between January 2010-December 2021. Website literature searches were conducted using Google Scholar and Baidu.

**Results:**

Sixty-nine publications and 3 clinical guidelines were included. Incidence ranged from 0 to 10.7 per 100 000 people per year in Australia, Japan, and Taiwan, and ranged from 6.3 to 24.70% among patients who underwent renal biopsy in mainland China. Prevalence and diagnosis rates ranged from 0 to 72.1% in mainland China, South Korea, Taiwan, Japan, and Australia. Mortality rates in mainland China, South Korea, and Japan varied widely. The top 3 commonly used therapies were angiotensin-converting enzyme inhibitor/angiotensin receptor blockers (0.9-99.6%), corticosteroids (3.5-100%), and immunosuppressants (1.6-85.5%) in Japan, mainland China, and South Korea. Patient quality of life was measured by different tools, and annual hospitalization costs ranged from $1 284.73 to $2 252.12 (2015–2018) in China.

**Conclusions:**

The prevalence of IgA nephropathy among the general population in select countries/regions is not commonly available, despite evidence from studies and clinical guidelines. In addition, it is observed across geographic regions that heterogeneity exists in prevalence rates, and large variations exist in treatment patterns. There is need to fill in these gaps to understand the contributing factors behind the differences through population-based, multi-center, and real-world studies.

**Supplementary Information:**

The online version contains supplementary material available at 10.1186/s12882-024-03555-5.

## Background

Immunoglobulin type A nephropathy (IgAN), also known as Berger’s disease, is a kidney disease caused by kidney deposition of immunoglobulin type A (IgA) complexes involving galactose-deficient IgA [1] and resulting in inflammatory tissue damage [2]. IgAN affects the kidneys by attacking the glomeruli and is characterized by persistent urinary abnormalities including microscopic hematuria, gross hematuria, and/or proteinuria [2, 3]. IgAN is the most common form of biopsy-proven primary glomerulonephritis (PGN) worldwide [3] and is one of the leading causes of chronic kidney disease (CKD) and end-stage renal disease (ESRD) [4].

Primary treatments for IgAN include angiotensin-converting enzyme inhibitor/angiotensin receptor blockers (ACEIs/ARBs), corticosteroids, and immunosuppressants [1, 4]. These treatments aim to address symptoms and manifestations of IgAN but not the underlying cause. Nearly one-third of IgAN patients develop ESRD within 10 years [5]. On average, patients with IgAN die 6 years earlier than the general population [6]. In addition, patients’ quality of life (QoL) is greatly impacted due to pain, fatigue, and poor mental health [4], and indirect caregiver burden is high due to time spent caring for patients who progress to ESRD. Thus, caregivers’ QoL and psychological well-being can also be negatively impacted [7].

IgAN prevalence is highest in Asia, intermediate in Europe and the US, and lower in African countries [8]. The overall global incidence is approximately 2.5 per 100,000 people per year [2]. A higher prevalence of IgAN is seen in countries where routine screening is practiced [4]. While geographic variations of IgAN have been studied previously [3, 9], few recent studies have focused on regional disease burden differences and treatment patterns in among IgAN patients across Asian countries/regions and Australia.

This review aimed to summarize the disease burden and treatment patterns of IgAN in select countries/regions in the Asia-Pacific region, specifically mainland China, Taiwan, South Korea, Japan, and Australia.

## Methods

### Data sources and search strategy

A targeted literature review (TLR) was conducted to identify relevant literature published from January 2010 to December 2021 for mainland China, Taiwan, South Korea, Australia, and Japan. The earliest year of publication was expanded from 2010 to 2001 to capture evidence more comprehensively on outcomes of interest. Medline and Embase were the primary databases for publications in English. For publications in local languages, WANFANG and China National Knowledge Infrastructure (CNKI) databases were searched for publications in Chinese, Korean Medical Database and Korean Information Service System (KISS) databases were searched for publications in Korean, and Scholarly and Academic Information Navigator (CiNii) was searched for publications in Japanese. Supplementary searches for clinical guidelines, conference proceedings, and websites of governmental and non-governmental organizations were conducted using Google, Baidu (for Chinese sources), and Naver (for Korean sources). Publications cited as references were also considered for screening.

Search terms included IgA nephropathy, Berger’s disease, incidence, prevalence, mortality, quality of life, cost, burden, and treatment. Observational studies, reviews, and registry studies were included in the search. Publications that reported prevalence, incidence, mortality, treatment patterns, guidelines, economic, and humanistic burden were included for data extraction. Search terms in English and local languages are listed in Supplementary Table S1.

### Study selection and data extraction

After the search was conducted and duplicates were removed, the title, abstract, and full texts of the remaining publications were screened. A second reviewer conducted the validation and finalization for publications to be included in the data extraction phase. During screening, the inclusion and exclusion criteria mainly focused on outcomes. Systematic reviews, observational studies including registry/database studies and other real-world studies, annual reports were considered for inclusion. Publications that reported evidence regarding epidemiology (incidence, prevalence, and mortality), humanistic and economic burden, and treatment patterns (treatment guidelines, duration, adherence, persistence, switching, and discontinuation) were included for data extraction. Studies that did not include outcomes of interest were excluded, as were studies with a small sample size (< 25). Strict predefined population, intervention, comparators, outcomes, and study design (PICOS) selection criteria and a Preferred Reporting Items for Systematic Reviews and Meta-Analyses (PRISMA) diagram were not used in this study. Study characteristics, patient characteristics, epidemiological outcomes, disease burden, and treatment patterns were extracted.

### Study quality assessment

All eligible studies went through a quality assessment (QA) using a recommended checklist, according to the Center for Reviews and Dissemination Guidance for Undertaking Reviews in Health Care recommendations [10]. Quality assessment was performed for all eligible articles by two reviewers. The checklist consisted of 9 items excluding basic information for the included studies. Because all publications included in this study were observational studies or reviews, only the non-randomized clinical trial checklist was used for observational studies.

## Results

Sixty-nine publications were included for this review, among which 38 were from mainland China (2015–2021) [11–48], 15 from Japan (2003–2021) [49–63], 10 from South Korea (2010–2020) [64–73], 3 from Taiwan (2014–2019) [74–76], and 3 from Australia (2001–2021) [77–79]; characteristics of the studies are shown in Supplementary Table S2. Approximately 83% the publications reported a retrospective study design (*n* = 57). For publications from mainland China, sample sizes ranged from 74 [37] to 4,367,829 [47], and male percentages ranged from 37.5% [17] to 97.3% [32]. For publications from Japan, sample sizes ranged from 52 [53] to 270,902 [63]; the male percentage ranged from 37.1% [58] to 56.96% [52]. For publications from South Korea, sample sizes ranged from 25 [64] to 5,114 [67]; the male percentage ranged from 36% [64] to 66.6% [73]. For publications from Taiwan, sample sizes ranged from 91 [75] to 7,073 [76]; the male percentage ranged from 45.9% [76] to 52.7% [75]. For publications from Australia, sample sizes ranged from 1,147 [78] to 2,457 [79]; the male percentage ranged from 60% [77] to 69.7% [79]. The Kidney Disease: Improving Global Outcomes (KDIGO) Clinical Practice Guideline [1] and 2 country-specific guidelines [80, 81] were also included for evidence on treatment patterns.

Sixty-eight journal articles were assessed for study quality (all details of the quality assessment are shown in Supplementary Table S3); one white paper was not included in the study quality assessment. Approximately 75% (51/68 articles) were deemed to be of good quality (i.e., without inherent flaws). Few studies reported the incidence/prevalence of IgAN directly and percentage of IgAN were extracted from included studies. The appropriateness of the statistical analysis conducted was not clear or not specified in 5 studies, as they did not define P values and the level of significance for all observations. Across studies, outcome measures were generally considered reliable. However, 33 articles stated that the results could be generalized to routine practice. In one case-control study, the similarity of both groups at the outset of the study was not clear.

### Incidence

Six publications provided evidence on IgAN incidence [30, 61, 63, 74, 77, 78] in Australia (*n* = 2), Japan (*n* = 2), mainland China (*n* = 1), and Taiwan (*n* = 1). Most were cross-sectional observational studies (*n* = 4), and sample sizes ranged from 156 [74] to 270,902 [63].

In Australia, IgAN incidence was estimated to be 1.41–10.5 per 100,000 people per year [77, 78]. According to Briganti 2001 [78], IgAN incidence in Australia was lowest (0.0 per 100,000 per year) among male children and highest (10.7 per 100,000 per year) among male adults [78]. In Japan, only 2 studies reporting incidence data among children were identified. Utsunomiya 2003 [63] reported an incidence rate of 4.5 per 100,000 per year among 270,902 junior high and elementary school students; Kajiwara 2020 [61] reported a rate of 3.3 per 100,000 per year among 60,816 junior high and elementary school students. Both publications collected urine samples through a school urinary screening system in students 6 to 15 years old. In mainland China, the incidence rate of IgAN was estimated to be 6.3% among elderly patients who underwent renal biopsy and 24.7% among non-elderly patients who underwent renal biopsy [30]. In Taiwan, IgAN incidence was estimated to be 5.5 per million per year among the general population (around 23.5 million between 2014 and 2016), based on 1,445 renal biopsy records from a registry database [74]. In general, IgAN incidence was higher in males (5.7 per 100,000 per year) compared with females (2.9 per 100,000 per year) [78]. IgAN incidence was not reported in Korean populations.

### Prevalence and diagnosis rate

IgAN prevalence among the general population was not reported in the included publications. But one cross-sectional study (*n* = 3,623) reported an IgAN prevalence rate of 0.03% among the general Chinese pediatric population [34]. Thirty-five publications were identified with diagnosis rates among 2 populations: patients who received renal biopsies and PGN patients [13, 14, 17–19, 21, 22, 24, 30, 31, 33–36, 39, 40, 43–48, 52, 59, 67–72, 74–76, 79]. Twenty-one publications were from mainland China [13, 14, 17–19, 21, 24, 30, 31, 33–36, 39, 40, 43–48], 6 from South Korea [67–72], 3 from Taiwan [74–76], 3 from Japan [52, 55, 59], and 1 from Australia [79]. The majority (88%) were cohort studies (*n* = 17) [13, 21, 31, 33, 35, 36, 39, 40, 43–46, 52, 68–71] and cross-sectional studies (*n* = 13) [14, 17–19, 21, 24, 34, 37, 47, 59, 67, 72, 74, 79], with the remainder being an annual report [76], a registry study [55] and a chart review [75]. Sample sizes ranged from 33 [70] to 43,67,829 [47].

**Fig. 1:**
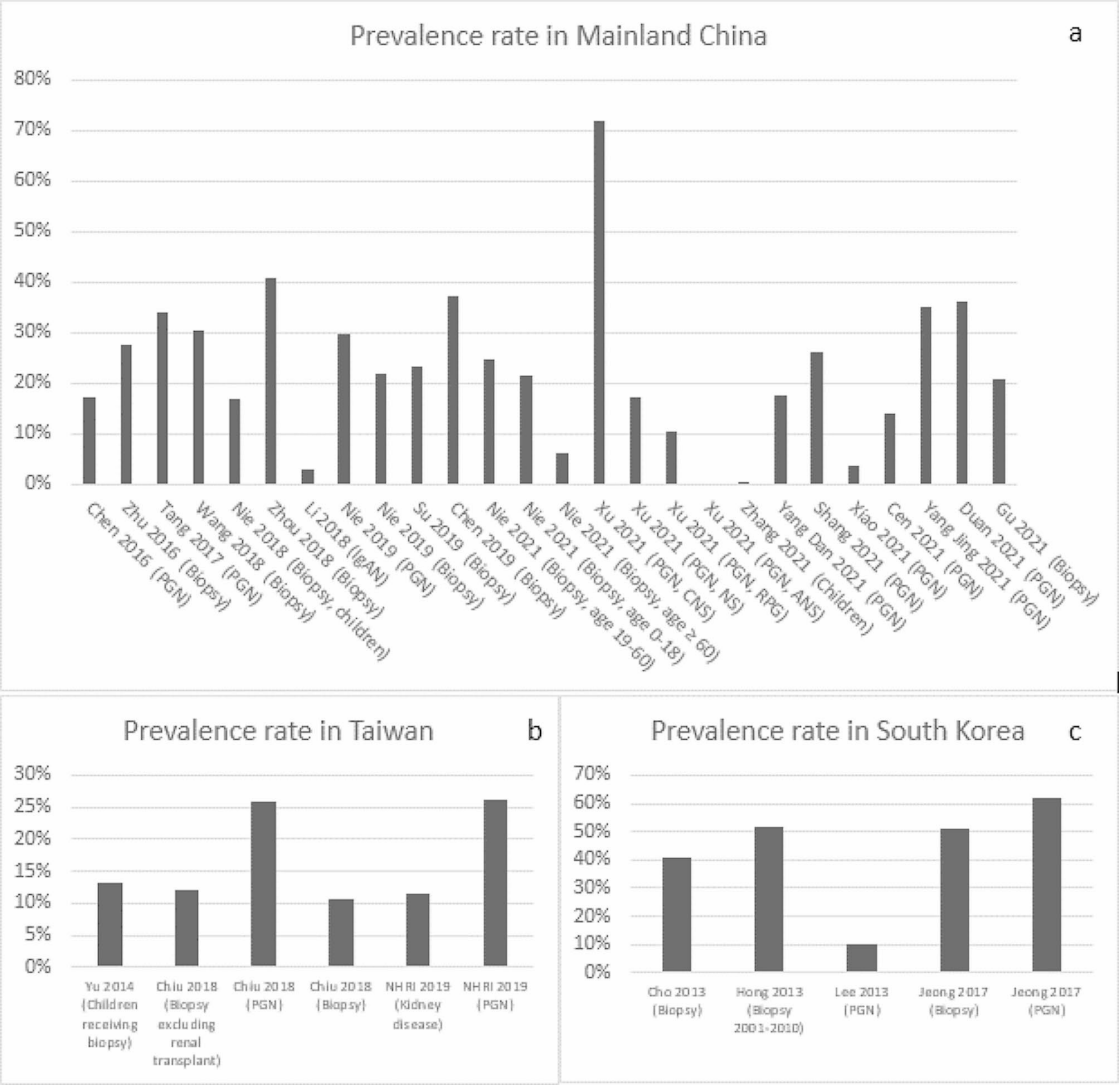
IgAN Prevalence in Mainland China, Taiwan and South Korea (Abbreviation: ANS, acute nephritic syndrome; CNS, chronic nephrotic syndrome; NHRI, National Health Research Institute & Taiwan Society of Nephrology; NS, nephritis syndrome(e; PGN, primary glomerulonephritis; RPG, rapidly progressive glomerulonephritis)

In mainland China, the mean diagnosis rate of IgAN was estimated to be 24.1% among patients undergoing renal biopsies (median: 23.0%; range: 6.3-40.9%) [13, 19, 21, 22, 24, 30, 46] and 27.3% (median: 27.9%; range: 0-72.1%) [14, 19, 21, 33, 36, 40, 43–45, 48] among PGN patients (Fig. 1a); The mean IgAN diagnosis rate was estimated to be 21.7% (median: 17.5%; 17-30.4%) among children who underwent renal biopsy [17, 18, 35]. In Taiwan, the mean diagnosis rate of IgAN was 12.1% (median: 12.2%; range: 10.8-13.2%) among patients undergoing renal biopsies [74, 75] and was reported similar (26%) among PGN patients [74, 76] (Fig. 1b). In South Korea, the mean diagnosis rate was 41% (median: 38.1%; range: 25.8-61.9%) among patients undergoing renal biopsies [67, 69, 71, 72] and around 51.6% (average of 51.3% and 51.9%) among PGN patients [68, 70] (Fig. 1c). In Japan, Hattori 2016 reported a mean estimated IgAN diagnosis rate of 23% (median: 22.9%) among CKD patients [59]. In addition, the reported IgAN diagnosis rate among patients who underwent renal biopsy was 31%, with 6.9% in patients aged 65 to 80 years old and 10.5% in patients aged 80 years or older [52, 55]. In Australia, Lee 2020 reported an IgAN diagnosis rate of 13% among patients undergoing renal biopsy [79].

### Disease progression and mortality

Among included studies, all-cause mortality was mainly reported as deaths due to ESRD. Seven publications from mainland China [23, 26–29, 41, 42], 7 from Korea [64–66, 68, 70, 71, 73], 4 from Japan [50, 51, 57, 62], and 1 from Taiwan [75] reported rate of progression to ESRD in IgAN. These studies varied in the definition of endpoint, patient characteristics, and follow-up duration. In China, the median rate of progression to ESRD was 4.1% [28] over 6 months, ranged from 1.3 to 15.8% (median: 1.3%) over 40–45 months [29, 41], ranged from 6.6 to 15% (median: 8.3%) over 4–10 years [23, 27, 42], and 33% over 15 years [42]. In Korea, the median rate of progression to ESRD ranged from 2.5 to 39.7% (median: 19%) from 60 to 100 months [64–66, 68, 70, 71, 73].

Regarding direct reports on mortality, in mainland China, 0.7% of adult IgAN patients progressed to death according to 1 study of 944 patients from 2003 to 2014 with a median follow-up of 4.2 years [23]. In South Korea, the median death rate was 5.3% (range: 4.4-5.9%) [65, 66, 68] for 1,364 IgAN patients with a median follow-up of 100 months. In addition, 2 publications reported a standard mortality ratio (expressed as the ratio between the observed and the expected number of deaths in the general population) of 1.43 (95% confidence interval:1.04–1.92) among 1,364 IgAN patients in relation to the general population [65, 68]. In Japan, IgAN mortality was estimated to be 0.3 per 100 person-years among non-smokers [51], 1.3 per 100 person-years among smokers [51] and 1.2 per 100 person-years among patients who received kidney replacement therapy [53] based on 2 retrospective studies [51, 53]. No mortality data was found among IgAN patients in Taiwan or Australia.

### Treatment patterns

Twenty publications [1, 11, 15, 26, 27, 29, 42, 49, 50, 54, 56–58, 60, 62, 64, 68, 71, 73, 81] and 3 clinical guidelines reported treatment patterns. Nine from mainland China [11, 15, 26–29, 32, 41, 42], 8 from Japan [49, 50, 54, 56–58, 60, 62], and 4 from South Korea [64, 68, 71, 73]. 80% publications were retrospective studies (*n* = 16) [11, 15, 26, 27, 29, 42, 49, 56–58, 60, 62, 64, 68, 71, 73]. Sample sizes ranged from 25 [64] to 2,283 [50]. The KDIGO [1] and 2 country-specific treatment guidelines, 1 from mainland China [80] and 1 from Japan [81], were identified. No treatment guidelines were identified in Taiwan, South Korea, or Australia.

The KDIGO guidelines (2021 version) provide treatment recommendations for adults and children with IgAN [1]. The guidelines state that the management of IgAN should be multifaceted, optimized with supportive care, and include ACEIs/ARBs as tolerated or allowed, control blood pressure, minimize cardiovascular risk, and adherence to lifestyle changes including dietary counseling, smoking cessation, weight control, and exercise, as appropriate. The guidelines provide specific treatment recommendations according to the variant forms of IgAN, the level of proteinuria, and high-risk rate for progression after maximal supportive care. The main treatment regimens include ACEIs and ARBs, immunosuppressants, cyclophosphamide, tonsillectomy, and lifestyle modification [1]. Similar to the KDIGO guidelines, the primary treatment recommendations in the Chinese 2017 guidelines for children with IgAN were glucocorticoids, immunosuppressants, and ACEIs/ARBs [80]. Japanese 2020 guidelines covered children and adults, with different treatment recommendations based on symptoms and subtype of IgAN (the subgroup classification for adults was based on estimated glomerular filtration rate and proteinuria; symptoms among children were classified as mild or severe) [81].

In mainland China, 6 studies investigated adult populations [15, 26, 28, 29, 32, 42] (Table 1) and 3 investigated pediatric populations [11, 27, 41] (Table 2). For drug usage among adult patients, ACEIs/ARBs had the largest median percentage at 66.7% (range: 38-90%) [15, 26, 28, 29, 32, 42], followed by steroids, with median of 36% (corticosteroids/prednisone/intravenous methylprednisolone injection, range: 10-100%) [15, 26, 28, 29, 32, 42] and immunosuppressants (including in combination with steroids), with median of 25.9% (cyclophosphamide, tacrolimus and tripterygium wilfordii, range: 1.6-72%) [15, 26, 28, 29, 32, 42]. Among pediatric patients, immunosuppressants (cyclophosphamide/mycophenolate /Tripterygium wilfordii /leflunomide) were the common drugs recommended, with a median of 64% (range: 1.7–72.2%) [11, 27, 41], followed by ACEIs/ARBs, with a median of 49.5% (range: 2.5-70%) [11, 27, 41] and steroids with a median of 45% (range: 25.3-69.3% as sum of oral prednisone and intravenous methylprednisolone) [11, 27, 41].

In South Korea, 3 publications on adult IgAN patients [64, 68, 71] (Table 1) and 1 publication among pediatric patients [73] (Table 2) were identified. Among adults, ACEIs/ARBs were the most common treatments (27.7-83.4%) [68, 71, 73], followed by ACEIs/ARBs and corticosteroid combinations (33.9%) [64] and corticosteroids alone (12.4-28.8%) [68, 71, 73]. Among pediatric patients, the frequency of immunosuppressant use was 50.2% [73].

In Japan, 7 publications reported IgAN treatment patterns among adults [50, 54, 56–58, 60, 62] (Table 1) and 2 publications [49, 54] among pediatric patients (Table 2). Among adults, ACEIs/ARBs were the most common treatment (25-99.6%) [50, 54, 56–58, 60, 62], followed by antiplatelet agents (58.1-96.8%) [54] and corticosteroid-immunosuppressant combination therapy (1.5-74%) [62]. Notably, the rate of administering steroid-immunosuppressant combination was only 1.5% in a retrospective cohort study that sampled 1,012 IgAN patients with a mean age of 32.96 ± 12 years [56]. Among pediatric patients, ACEIs/ARBs were the most frequently administered treatments (0.9-95.7%) [49, 54], followed by antiplatelet agents (range: 1.2-82.6%) [49, 54] and immunosuppressants (range: 4.6-68.5%) [49]. The frequency of administering treatments varied greatly across different subgroups. For example, the frequency of administering ACEIs/ARBs ranged from 0.9% for the diffuse mesangial proliferation subgroup (*n* = 108) to 50.9% for the focal mesangial proliferation subgroup (*n* = 173) in 1 retrospective study in Japanese children with IgAN from 1990 to 2004 [49]. Tonsillectomy or tonsillectomy combined with steroid was mostly reported in Japanese studies, with frequencies ranging from 1 to 66.2% across publications (Table 1). This is in accordance with the KDIGO 2021 guidelines’ evidence that supports the routine use of tonsillectomy in Japanese high-risk patients with IgAN [1]. No publications reporting IgAN treatment patterns were identified for Taiwan or Australia.

**Table 1:** Treatment patterns among adult patients across select countries/regions

Author year	Cohort age ^a^	Sample size	ACEI/ARBs (%)	Other therapies (%) ^e^
**Mainland China**				
Wen 2021 [29]	32.7 ± 11.1 (Male); 34.7 ± 10.8 (Female)	1096	38	Steroid (36.1), immunosuppressant (25.9)
Zhu 2021 [32]	35.5 ± 9.1	102	78.4	Steroid (100), tonsillectomy (27.5), intravenous methylprednisolone (15.7), plasma exchange (8.8)
Liu 2020 [28]	37.76 ± 12.1	246	69.1	CTX + steroid (55.7), steroid (52.8)
Tian 2020 [26]	33.8 ± 11.6	172	55.8	Steroid (32.6), steroid + immunosuppressant (9.9), immunosuppressant (7.6)
Zhou 2017 [15]	36 (28–43) ^b^	375	66.7	Calcium channel blockers (29.1), corticosteroid (20), β-blocker (14.9), allopurinol (6.7), α-blocker (3.7), immunosuppressant (1.6), diuretics (0.8)
Le 2011 [42]	30.6 ± 9.4	1126	90	Tripterygium glycosides (72), other immunosuppressants (13.5), prednisolone (10)
**Japan**				
Miyabe 2021 [62] O-grade I ^c^	30 (24–42)	260	25	Steroid + immunosuppressant (30)
Miyabe 2021 [62] O-grade II ^c^	30 (24–40)	525	35	Steroid + immunosuppressant (54)
Miyabe 2021 [62] O-grade III ^c^	34.5 (27–46.3)	86	49	Steroid + immunosuppressant (74)
Kaihan 2017 [60] (*n* = 86)	36 (26–46)	86	84	Steroid (66), tonsillectomy (33), immunosuppressant (8)
Oshima 2015 [58] 20–39 years	27 (23–32)	162	30.5	Antiplatelet agents (55), steroid (37.8), tonsillectomy + steroid (18.7), immunosuppressant (2.5), tonsillectomy (1.7)
Oshima 2015 [58] 40–59 years	47 (43–52)	162	59	Antiplatelet agents (68.9), steroid (30.4), tonsillectomy + steroid (13.7), immunosuppressant (2.5), tonsillectomy (1.9)
Oshima 2015 [58] ≥ 60 years	64.5 (62–69)	31	67.7	Antiplatelet agents (58.1), steroid (51.6), immunosuppressant (3.2)
Sato 2015 [57]	42 (range, 18–73)	198	60.6	Steroid (59.1), immunosuppressant (59.1), tonsillectomy (1)
Moriyama 2014 [56]	33 ± 12	1,102	28.9	Antiplatelet agents (59.3), steroid (26.9), tonsillectomy + steroids (11.7%), tonsillectomy (1.5), steroid + immunosuppressant (1.5)
Matsuzaki 2013 [54]	NR	284	99.6	Antiplatelet agents (96.8), tonsillectomy + steroids (66.2), steroid pulse monotherapy (56), oral steroid (54.9%)
Goto 2009 [50]	32.1 (20.7–46.9)	2283	28.2	Steroid (34.5), immunosuppressant (10.6)
**South Korea**				
Kee 2017 [71]	38.5 ± 13.1	574	83.4	Steroid (18.5), immunosuppressant (4)
Lee 2013 [68] ^d^	35 (26–46)	1009	70.7	Steroid (12.4), immunosuppressant (3.4)
Lee 2010 [64]	38.1	177	27.7	ACEI/ARB + steroid (33.9), steroid (28.8), no treatment (8.5)

Abbreviations: ACEI/ARB, angiotensin-converting enzyme inhibitor/angiotensin receptor blocker; NR, not reported

a. Data is expressed as mean ± SD or median (then interquartile range)

b. Patient > 14 years were included in the study

c. O-grade is based on the Oxford classification system. Patients > 16 years were included in the study

d. Patients > 15 years were included in the study

e. Immunosuppressants were reported as a general type or specific drug (e.g., leflunomide, mycophenolate mofetil, tripterygium wilfordii, cyclophosphamide tacrolimus, etc.)

**Table 2:** Treatment patterns among pediatric patients across select countries/regions

Author year	Cohort age ^a^	Sample size	ACEI/ARBs (%)	Other therapies (%)
**Mainland China**				
Zhao 2021 [41]	8.4 ± 3.2	79	2.5	Steroid + immunosuppressant (72.2), steroid (25.3)
Wu 2020 [27]	13.7 ± 3.7	1243	70	Immunosuppressant (64), steroid (45), steroid + immunosuppressant (19)
WGNSSDTCRD [11]	10 (NR)	1417	49.5	Oral prednisone (47.5), intravenous methylprednisolone (21.8), CTX (19.6%), tripterygium wilfordii (15.2), MMF (6.3), leflunomide (1.7%), tonsillectomy (0.3)
**Japan**				
Matsuzaki 2013 [54]	NR	92	95.7	Antiplatelet agents (82.6), tonsillectomy + steroid (38), intravenous steroid (33.2%), oral steroid (30.4)
Yata 2008 [49] Children with DMP	10.9 (2.5–19.6) ^b^	108	0.9	Immunosuppressant (68.5), steroid (23.1), antiplatelet agents (6.5%), no treatment (0.9)
Yata 2008 [49] Children with FMP	10.9 (2.5–19.6) ^b^	173	50.9	No treatment (13.2), steroid + immunosuppressant (4.6), steroid (3.5%), antiplatelet agents (1.2)
**South Korea**				
Suh 2020 [73]	11.41 ± 3.9	1154	31.9	Immunosuppressant (50.2)

Abbreviations: ACEI/ARB, angiotensin-converting enzyme inhibitor/angiotensin receptor blocker; CTX, cyclophosphamide; DMP, diffuse mesangial proliferation; FMP, focal mesangial proliferation; MMF, mycophenolate mofetil; WGNSSDTCRD, Working Group for National Survey on Status of Diagnosis and Treatment of Childhood Renal Diseases; NR, not reported

(a) Data are expressed as mean ± SD or median (then interquartile range). (b) Baseline sample (*n* = 500)

### Humanistic burden

Four publications in China reported QoL, measured by the 36-Item Short Form Health Survey (SF-36) [16, 25], Daily Living Ability Rating Scale (DLARS) [37], and QoL scale (QOLs) combined with Self-Rating Anxiety Scale (SAS) and Self-Rating Depression Scale (SDS) [38]. SF-36 scores reflect physical and mental health based on 8 health concepts, including physical and social functioning, role limitations due to physical and emotional problems, mental health, vitality, bodily pain, and general health (GH) perception [82]. Two publications evaluated the effects of individualized nursing intervention (INI, one improved nursing intervention which costs more time than routine nursing intervention [RNI]) on the psychological mood and QoL among IgAN patients [16, 25]. There were two subgroups, the patients in the control group received RNI and patients in the intervention group received INI [16, 25]. The mean GH score was 32.16 [16] among total IgAN patients (*n* = 98; mean age: 32.74 years; male percentage: 50%) in 2017 and 80.15 increasing from 69.93 at baseline [25] after intervention among total IgAN patients (*n* = 84; mean age: 33.57 years; male percentage: 60.7%) in 2019. In both publications, the intervention groups had higher mean GH scores than that in the control groups (39.47 vs. 24.84 [16] and 85.73 vs. 74.56 [25], respectively). Two other prospective studies assessed the effect of INI for IgAN patients [37, 38]. Results showed that both mean DLARS and QOLs scores were higher among the intervention group compared to the control group (88.5 vs. 75.7 and 39.5 vs. 24.8, respectively) [37, 38]. SAS and SDS scores were also evaluated by Qi 2021 [38], the mean SAS score decreased more in the intervention group (49.2 ± 6.3 decreased from 62.1 ± 5.8) than that in the control group (57 ± 4.9 decreased from 62.4 ± 6.1) from baseline. Similarly, the mean SDS score decreased more in the intervention group (43.3 ± 5.2 decreased from 56.2 ± 6) than in the control group (52.6 ± 6.4 decreased from 57 ± 6.2) from baseline [38].

### Economic burden

No publications reported indirect costs, but 3 retrospective studies reported hospitalization costs for IgAN patients in China (see Supplementary Figure S1) [12, 20, 47]. Hospitalization cost per patient per year is ¥14,900 ($2,252.12; exchange rate of Chinese Yuan [CNY] and US dollar in 2018 was 6.616 [83]) as reported by Zheng 2018 [20], and between ¥9,618 ($1,532.26; exchange rate of CNY and US dollar in 2015 is 6.227 [83]) and ¥10,019 ($1,608.96) as reported by Peng 2015 [12]. One large database study covering 54.1% of tertiary hospitals in 31 Chinese provinces from 2010 to 2015 reported a hospitalization cost of ¥8,000/$1,284.73 (¥6,000-¥12,000) [47]. Drug costs accounted for 28.39% of total hospitalization costs, followed by diagnostic testing costs [12]. Length of stay per patient per year in China ranged from 10 to 14.3 days across 3 publications [12, 20, 47].

## Discussion

To our knowledge, this is the first TLR to summarize the evidence on IgAN disease burden and treatment patterns in mainland China, Taiwan, South Korea, Japan, and Australia. The findings of this review revealed evidence gaps in IgAN epidemiology and humanistic and economic burden. No incidence data was identified in South Korea; no mortality data was identified in Taiwan and Australia; no country/region-specific treatment guidelines were found for Taiwan, South Korea, or Australia; no evidence on treatment patterns from the publications was identified for Taiwan or Australia; and no humanistic burden or economic data was identified except for mainland China.

The IgAN incidence rates among Japanese, Taiwanese, and Australian populations ranged from 0 to 10.7 per 100,000 people per year, higher than the incidence rate reported in a recent systematic literature review (SLR) by Kwon 2021 [84] (1.29 per 100,000 people per year). Kwon 2021 [84] is an SLR focusing on US epidemiology, health-related QoL, and the economic burden of IgAN (the included studies were published from January 2010 to June 2020), similar to our study’s objective. Incidence rates among children and teenagers (0-4.5 per 100,000 per year) were similar to the incidence rate in Venezuela (0.03 per 100,000 per year) [85] and in Italy (0.31 per 100,000 per year) [86]. The overall prevalence and diagnosis rates of IgAN were similar across selected countries/regions. The diagnosis rates in this review differed from those found in PGN patients and patients who received renal biopsy in Kwon 2021 [84]; diagnosis rates of IgAN from our results were higher in PGN patients compared with patients who received renal biopsies since renal biopsies were often performed on PGN patients before diagnosis. This applied to both adult and pediatric populations. Compared to the US population in Kwon 2021 [84], the diagnosis rate among PGN populations in this review was higher (26-72.1% vs. 9.4-19.7%). The diagnosis rate among populations with renal biopsies was also higher (6.3-61.9% vs. 6.3-14.3%). Notably, though not covered by this review, the pathological profile such as Oxford Classification/MEST classification could also shed light upon disease burden, which could be further explored by future studies.

IgAN treatments primarily consisted of ACEIs/ARBs, and high utilization of steroids was found despite mixed evidence on their benefits and safety. There is limited data on IgAN treatment patterns from Taiwan and Australia. Among the publications that reported treatment patterns, few specified drugs’ generic names. The primary treatment patterns reported among select countries/regions in this study are similar to those in US as reported by Kwon 2021 (frequently used therapies were immunosuppressives, corticosteroids, and ACEIs/ARBs) [84]. Immunosuppressives were used more by children than adults based on data from mainland China, South Korea, and Japan. According to the KDIGO guideline regarding glomerular diseases, the immunosuppressive therapies including azathioprine, cyclophosphamide, calcineurin inhibitors, and rituximab are not recommended for treating IgAN. Mycophenolate mofetil is recommended in Chinese patients and tonsillectomy is recommended to be used in Japanese IgAN patients [1]. Only Chinese studies reporting SF-36 scores and other metrics were identified. Therefore, more studies on QoL in IgAN patients and caregivers in other regions are warranted.

Evidence of economic burden was identified only from studies in mainland China; Li 2018 was one retrospective national inpatient database study, which included the major hospitals that covers multiple geographic locations [47], other two studies used the data from one hospital. The mean cost per patient per year reported by Li 2018 is $1,284.73, while one Canadian retrospective study for costs and healthcare resource utilization reported a mean outpatient medication cost per patient per year of Canadian dollar (CAD) $221 in 2016 [87]. To control medical costs, hospitals in China are undergoing clinical pathway optimization programs [12].

Publications reported heterogeneous sample populations where IgAN prevalence/diagnosis rates were evaluated. Among 22 publications that reported IgAN prevalence/diagnosis rates, 15 measured IgAN prevalence for patients who underwent renal biopsy and 9 measured IgAN prevalence for patients diagnosed with PGN. Heterogeneity in IgAN prevalence/diagnosis rates may be attributed to differences in study years, patient race/ethnicity, patient age, treatment method, risk factors, diagnosis, and follow-up duration. Other study design–related factors that could introduce bias include sample size and gender composition.

Finally, differences in IgAN prevalence across regions should be noted. County/region-specific healthcare infrastructure and policies influence the epidemiological evidence of IgAN. systematic urine screening programs among individuals with asymptomatic, persistent microscopic hematuria with/without mild proteinuria are commonly implemented in certain countries/regions. These programs facilitate detection of IgAN patients who would otherwise receive a delayed diagnosis or none at all. Countries/regions where screening programs are performed may therefore have higher reported IgAN prevalence. Screening programs play a crucial role in early diagnosis and early treatment [88].

To our knowledge, this is the first TLR for IgAN in mainland China, Taiwan, South Korea, Japan, and Australia. However, several limitations should be noted. Due to the targeted nature of this review, the search focused on the most relevant literature, and the publications included in this study were prioritized, which potentially have led to an incomplete picture of IgAN-related epidemiology, treatment patterns and disease burden. Across included publications, the sample sizes varied widely and were not always reported. Additionally, this TLR did not weigh the data from included publications; therefore, biases should be considered when comparing outcomes. Studies came from primarily single institutions, and national-level data was not always available for the selected countries/regions. Moreover, this review only covered select Asia-Pacific countries/regions; future reviews and studies in other countries and regions within Asia-Pacific are therefore warranted. Despite these limitations, the evidence gathered in this literature review may help provide a preliminary understanding of the disease burden of IgAN in the Asia-Pacific region.

This TLR summarized evidence on Immunoglobulin type A nephropathy (IgAN) prevalence, treatment patterns, and humanistic and economic burden. Our results suggest that despite the overall scarcity of information in general, evidence on disease burden and treatment patterns has been reported by some studies and several clinical guidelines. The prevalence of IgAN among the general population is not commonly available, while that among patients receiving renal biopsies and diagnosed with PGN is more frequently reported. Heterogeneity in prevalence rates across geographic regions might be explained by differences in initial diagnosis in some regions due to variation in local screening policy and disease management. There is a need to understand how the disease progression differs by those practices. Treatment patterns have been reported mainly in studies from some Asia areas, but geographic variations are noticeable. There is also a need to generate more evidence to shed light upon the possible explanation to the differences in the treatment patterns across geographic regions. In sum, more real-world studies at national levels across select countries/regions are warranted to fill the evidence gaps, particularly regarding incidence, humanistic burden, and economic burden.

## Conclusion

The prevalence of IgA nephropathy among the general population in select APAC countries/regions is not commonly available, despite evidence from studies and clinical guidelines. In addition, it is observed across geographic regions that heterogeneity exists in prevalence rates, and large variations exist in treatment patterns. Future studies are needed to fill in these gaps to understand the contributing factors behind the differences through population-based, multi-center, and real-world studies.

### Electronic supplementary material

Below is the link to the electronic supplementary material.

Supplementary Material 1

## Data Availability

All data generated or analyzed during this study are included in this published article [and its supplementary information files].

## Abbreviations

ACEI: Angiotensin-converting enzyme inhibitor
ANS: Acute nephritic syndrome
APAC: Asia Pacific
ARBs: Angiotensin receptor blockers
CAD: Canadian dollar
CKD: Chronic kidney disease
CNKI: China National Knowledge Infrastructure
CNS: Chronic nephrotic syndrome
CNY: Chinese Yuan
CTX: Cyclophosphamide
DLARS: Daily Living Ability Rating Scale
DMP: Diffuse mesangial proliferation
EMBASE: Excerpta Medica Database
ESRD: End-stage kidney failure
FMP: Focal mesangial proliferation
GH: General health
INI: Individualized nursing intervention
KDIGO: The Kidney Disease: Improving Global Outcomes
KISS: Korean Information Service System
MMF: Mycophenolate mofetil
NR: Not reported
NS: Nephritis syndrome
PGN: Primary glomerulonephritis
PICOS: Population, intervention, comparators, outcomes, and study design
PRISMA: Preferred Reporting Items for Systematic Reviews and Meta-Analyses
QA: Quality assessment
RNI: Routine nursing intervention
RPG: Rapidly progressive glomerulonephritis
SAS: Self-Rating Anxiety Scale
SD: Standard deviation
SDS: Self-Rating Depression Scale
SF-36: 36-Item Short Form Health Survey
SLR: Systematic literature review
TLR: Targeted literature review
TSN: Taiwan Society of Nephrology
WGNSSDTCRD: Working Group for National Survey on Status of Diagnosis and Treatment of Childhood Renal Diseases
