# Supplementary material for: Review on epidemiology, disease burden, and treatment patterns of IgA nephropathy in select APAC countries
Source: BMC Nephrol. 2024 Apr 16;25:136. doi: 10.1186/s12882-024-03555-5 (PMC11021013; doi:10.1186/s12882-024-03555-5)
Supplement: Supplementary file 1 — Supplementary Material 1 [file 12882_2024_3555_MOESM1_ESM.docx]

# Supplementary Material

Table S1. Search terms in English, Chinese, Korean, and Japanese

| **Topic** | English terms | Chinese terms | Korean terms | Japanese terms |
| --- | --- | --- | --- | --- |
| Disease | IgA nephropathy | IgA肾病 | IgA신증 | IgA腎症  IgA腎炎  バージェ病 |
|  | Berger's disease | 伯杰氏病 | 버거씨병/Berger씨 병 | Berger病  ベルジェ病 |
| Epidemiology | Incidence | 发病率  患病率  发病/患病率  流行病  死亡  危险因素  生存/存活率  临床指南/治疗指南  临床实践  治疗模式  专家共识  真实世界  剂量  疗程  依从性  持续率/性  治疗转换  *线治疗  停药率  门诊患者  住院病人/患者  住院  住院时间/住院天数  检查/检测  负担  成本/费用  支出/花费  生活质量  效用  照顾/照护  生产力 | 발생률  발생률  유병률  이환율  역학  사망률  위험인자  치료지침/진료지침  진료  치료 방식  전문가적 공감  실세계  정량  치료기간  (복용)순응도/부착도  (복용)지속도  치료 스위치  *차/선 치료  (치료)중단  외래 환자  입원환자  입원  입원기간  검사하다/검사/찾기  부담  비용  지출  삶의 질  유용성  간병  생산성 | 疫学/患者数  発生/発症率/有病率  罹患/病態/死亡  危険因子  リスクファクター  リスク因子  生存  指針  ガイドライン  治療指針/診療指針  治療ガイド  診療ガイド  治療  実践  リアルワールド  RWD/実世界  投与量  治療期間  遵守  アドヒアランス  コンプライアンス  継続  パーシスタンス  切り替え  治療ライン  用量  中止/中断  入院/入院期間  検査/検診  テスト  医療費/自己負担/費用/支出/負荷  生活の質/QOL /HQL/HQOL/HRQOL  合併症/障害 異常  効用  労働損失/生産性  間接費用  介護/負担 |
|  | Prevalence |  |  |  |
|  | Morbidity |  |  |  |
|  | Epidemiology |  |  |  |
|  | Mortality |  |  |  |
|  | Risk factor |  |  |  |
|  | Survival |  |  |  |
| Treatment patterns | Treatment guideline |  |  |  |
|  | Treatment practice |  |  |  |
|  | Treatment pattern |  |  |  |
|  | Expert consensus |  |  |  |
|  | Real-world |  |  |  |
|  | Dosage |  |  |  |
|  | Treatment duration |  |  |  |
|  | Adherence |  |  |  |
|  | Persistence |  |  |  |
|  | Treatment switch |  |  |  |
|  | Line of therapy |  |  |  |
|  | Discontinue* |  |  |  |
| Resource utilization | Outpatient |  |  |  |
|  | Inpatient |  |  |  |
|  | Hospitalization |  |  |  |
|  | Length of stay |  |  |  |
|  | test OR examine |  |  |  |
| Burden of illness | Burden |  |  |  |
|  | Cost |  |  |  |
|  | Expenditure |  |  |  |
|  | Quality of life/ life quality |  |  |  |
|  | Utility |  |  |  |
|  | Caregiver |  |  |  |
|  | Productivity |  |  |  |

Table S2. Study characteristics of all included studies by country

| Author Year | Country | | Study design | Perspective | Targeted population | Study year | Sample size | Mean age (SD) | Gender：Male (%) |
| --- | --- | --- | --- | --- | --- | --- | --- | --- | --- |
| Briganti 2001 [1] | Australia | | Cross-sectional | Retrospective | Patients with renal biopsies of histological diagnosis as glomerulonephritis | 1995-1997 | 1,147 | NA | NA |
| Jegatheesan 2016 [2] | | Australia | Cross-sectional | Retrospective | Native adult patients with glomerulonephritis -related renal biopsies | 2002-2011 | 2,048 | 48 (17) | about 60% |
| Lee 2021 [3] | | Australia | Cross-sectional | Retrospective | Patients undergoing renal biopsy | 2014-2019 | 2,457 | 48.4 (range: 18-93) | 69.7% |
| Chiu 2018 [4] | | China (Taiwan) | Cross-sectional | Prospective | Patients undergoing kidney biopsy | 2014-2016 | 1,445 | 48.4 (16.6) | 53.8% |
| NHRI & TSN 2019* [5] | | China (Taiwan) | Annual Report | NA | Patients with kidney disease, IgAN subgroup | 2019 | 7,073 | 41.9 (IQR: 31.5–54.1) | 45.9% |
| Yu 2014 [6] | | China (Taiwan) | Chart review | Retrospective | Kidney biopsy-Children | 2005-2009 | 91 | 10.9 (4.4) | 52.7% |
| Goto 2009 [7] | | Japan | Cohort | Prospective | IgAN patients | 1995-2005 | 2,283 | median 32.1 (range 20.7–46.9) | 49.70% |
| Hattori 2016 [8] | | Japan | Cross-sectional | Retrospective | Chronic kidney disease patients | 2014 | 3,138 | Range: > 20 | NA |
| Kaihan 2017 [9] | | Japan | Cohort | Retrospective | Adult (age >18 years) IgAN patients | 2001-2015 | 86 | Median: 36 (IQR: 26-46) | 51% |
| Kajiwara 2020 [10] | | Japan | Cohort | Prospective | General students aged 6-15 years | 2012-2018 | 14,606 | Range: 6-15 | NA |
| Komatsu 2013 [11] | | Japan | Cohort | Retrospective | IgAN patients who reached end-stage kidney disease after renal replacement therapy | 1981-2010 | 52 | 35.5 (12.8) | 50% |
| Matsuzak 2013 [12] | | Japan | Cross-sectional | Prospective | Hospital-treated IgAN patients | 2008 | 376 hospitals | NA | NA |
| Miyabe 2021 [13] | | Japan | Cohort | Retrospective | IgAN patients | 1974-2015 | 871 | 31 (range: 24–41) | 41% |
| Moriyama 2014 [14] | | Japan | Cohort | Retrospective | IgAN patients | 1974-2011 | 1,012 | 32.96 (12.0) | 40.51% |
| Oshima 2015 [15] | | Japan | Cohort | Retrospective | IgAN adult patients proven by renal biopsy | 1992-2011 | 600 | AAG subgroup: 64.5 (range: 62.0–69.0); MAG subgroup: 47.0 (range: 43.0–52.0); YAG subgroup: 27.0 (range: 23.0–32.0) | AAG: 45.2% MAG: 38.3%; YAG: 37.1% |
| Sato 2015 [16] | | Japan | Cohort | Retrospective | IgAN patients | 1980-2001 | 198 | HG1 subgroup: 42 (range: 18–72);  HG2 subgroup: 43 (range: 21–73);  HG3/4 subgroup: 49 (range: 21-70) | 47.98% |
| Sugiyama 2013 [17] | | Japan | Registry | Prospective | Patients included in the  J-RBR and J-KDR (J-RBR/J-KDR)- biopsies or cases | 2009-2010 | 7,442 | NA | NA |
| Utsunomiya 2003 [18] | | Japan | Cohort | Retrospective | Pediatric population under  15 years | 1983-1999 | 270,902 | Range: 4-16 | NA |
| Yamamoto 2010 [19] | | Japan | Cohort | Retrospective | IgAN patients | 1992-2005 | 971 | Non-/Past Smokers: 31 (range: 22-46); Current Smokers: 34 (range: 25-48) | Non-/Past Smokers: 38.4%; Current Smokers: 63.7% |
| Yata 2008 [20] | | Japan | Cohort | Retrospective | Children with IgAN | 1976-2004 | 500 | Median: 10.9 (range: 2.5-19.6) | 55.80% |
| Yokoyama 2012 [21] | | Japan | Cohort | Retrospective | Elderly patients (over 65 years old) who had undergone native renal biopsy | 2007-2011 | 2,802 | Age groups: 20-6, 65+ | 56.96% |
| Bae 2013 [22] | | Korea | Cohort | Retrospective | IgAN patients undergoing kidney biopsies (age >15 years) | 1981-2010 | 2414 | 35.9 (range: 15-91) | 0.5742 |
| Cho 2013 [23] | | Korea | Cross-sectional | Retrospective | Children | 1999-2008 | 5,114 | 10.16 | 55.6% |
| Jeong 2017 [24] | | Korea | Cohort | Retrospective | Adult primary IgAN inpatient | 1990-2013 | 33 | 44 | 48.5% |
| Kee 2017 [25] | | Korea | Cohort | Retrospective | IgAN patients | 2005-2013 | 574 | 38.5 (13.1) | 52.3% |
| Lee 2010** [26] | | Korea | Cohort | Retrospective | IgAN with ARF | 1997-2006 | 25 | 44.02 (17.14) Range: 18-74 | 36.0% |
|  |  | Korea | Cohort | Retrospective | IgAN with CRF | 1997-2006 | 58 | 41.24 (13.16) Range: 18-78 | 67.2% |
|  |  | Korea | Cohort | Retrospective | IgAN patients with normal renal function | 1997-2006 | 94 | 34.51 (12.26) Range: 18-68 | 53.1% |
|  |  | Korea | Cohort | Retrospective | IgAN patients | 1997-2006 | 177 | 38.05 | 53.1% |
| Lee, Kim 2012 [27] | | Korea | Cohort | Retrospective | IgAN patients | 1979-2008 | 1,364 | 33 (IQR: 25–45) | 50.0% |
| Lee 2012 [28] | | Korea | Registry | Retrospective | IgAN patients >15 years | 1979-2008 | 1,374 | Range: >15 | NA |
| Lee 2013** [29] | | Korea | Cohort | Retrospective | IgAN patients | 1979-2008 | 1,009 | 35 (range: 26–46) | 49.1% |
|  |  | Korea | Cohort | Retrospective | Patients undergoing kidney biopsies | 1979-2008 | 1,943 | NA | NA |
| Shin 2017 [30] | | Korea | Cross-sectional | Retrospective | Patients undergoing kidney biopsies | 1992-2011 | 818 | 37.2 (range: 18-83) | 55% |
| Suh 2020 [31] | | Korea | Cohort | Retrospective | IgAN patients | 1985-2015 | 1,154 | 11.41 (3.87) | 66.6% |
| Cai 2019 [32] | | Mainland China | Cohort | Retrospective | Primary IgAN (age >18 years) | 2003-2014 | 944 | 36 (12) | 51.0% |
| Cen 2021 [33] | | Mainland China | Cohort | Retrospective | Patients who underwent renal biopsy | 2013-2020 | 630 | 44.08 (16.08) | 57.6% |
| Chen 2016 [34] | | Mainland China | Cohort | Retrospective | Patients with crescentic glomerulonephritis | 2003-2013 | 528 | 37.6 (16.4) | 39.4% |
| Chen 2019 [35] | | Mainland China | Cross-sectional | Retrospective | Patients with renal insufficiency | 2008-2017 | 969 | Median: 48 | 55.1% |
| Duan 2021 [36] | | Mainland China | Cohort | Retrospective | Renal biopsy cases | 1986-2019 | 10,684 | NA | 52.4% |
| Feng 2021 [37] | | Mainland China | Cohort | Retrospective | Chronic kidney disease patients | 2015-2019 | 1,002 | Median: 7 (range: 0-18) | 63.0% |
| Gu ** 2021 [38] | | Mainland China | Cohort | Retrospective | Renal biopsy cases, Gui hospital | 2014-2018 | 1,370 | Gui hospital: 41.48 (13.90) | 49.5% |
|  |  | Mainland China | Cohort | Retrospective | Renal biopsy cases, Ji hospital | 2014-2018 | 1,370 | Ji hospital: 41.12 (13.4) | 54.1% |
| Huang 2019 [39] | | Mainland China | Cohort | Retrospective | Adult primary IgAN inpatient | 2017-2018 | 84 | 34.11 (2.58) | 60.7% |
| Le 2011 [40] | | Mainland China | Case series | Retrospective | Adult primary IgAN inpatient | 1989-2005 | 1,126 | 30. 6 (9. 4) | 49.5% |
| Li 2018** [41] | | Mainland China | Cross-sectional | 2010-2012 retrospective; 203-2015 prospective | Patients with the presence of nephrotic syndrome or glomerulonephritis | 2010-2015 | 43,677,829 | NA | NA |
|  |  | Mainland China | Cross-sectional | 2010-2012 retrospective; 203-2015 prospective | Patients with IgAN | 2010-2015 | 11,569 | 37.7 (14.2) | 51.1% |
| Liu 2021 [42] | | Mainland China | Cohort | Retrospective | Biopsy-confirmed IgAN patients | 2012-2018 | 246 | 37.76 (12.05) | 44.3% |
| Lu 2017 [43] | | Mainland China | Cohort | Retrospective | IgAN | 2011-2015 | 98 | 32.74 (10.64) | 50.0% |
| Lu 2021 [44] | | Mainland China | Cohort | Prospective | IgAN patients | 2019-2020 | 74 | 31.81 (1.6) | 54.1% |
| WGNSSDTCRD 2013 [45] | | Mainland China | Cross-sectional | Retrospective | Children with IgAN | 2008-2011 | 1,417 | Median: 10.0 | 67.2% |
| Nie 2018 [46] | | Mainland China | Cross-Sectional Survey | Retrospective | Children (0-18 years) undergoing kidney biopsy | 2004-2014 | 7,962 | 13.5 (4.1) | NA |
| Nie 2019 [47] | | Mainland China | Cross-sectional | Retrospective | Patients undergoing kidney biopsy | 2008-2017 | 4,910 | 42.6 (15.7) range: 7-84 | 54.0% |
| Nie 2021 [48] | | Mainland China | Cohort | Retrospective | Patients <60 years undergoing kidney biopsy | 2006-2020 | 5,923 | 38.2 (12.8) | 52.6% |
| Pan 2021** [49] | | Mainland China | Cohort | Prospective | Nephrotic patients, NSPN-IgAN | 2013-2018 | 174 | Range: > 50 | 44.4% |
|  |  | Mainland China | Cohort | Prospective | Nephrotic patients, NSPN-IMN | 2013-2018 | 174 | Range: > 50 | 50.5% |
| Peng 2015 [50] | | Mainland China | Chart review | Retrospective | IgAN-control group | 2010-2013 | 298 | 45.8 | 45.3% |
| Qi 2021 [51] | | Mainland China | Cohort | Prospective | Biopsy-confirmed IgAN patients, intervention group | 2018-2019 | 76 | 32.87 (1.67) | 56.8% |
| Shang 2021 ** [52] | | Mainland China | Cohort | Retrospective | Biopsy-confirmed PGN patients, Yubei area | 2008-2019 | 3,985 | Yubei area: 39.5 (16.9) | 55.0% |
|  |  | Mainland China | Cohort | Retrospective | Biopsy-confirmed PGN patients, Hainan area | 2008-2019 | 3,985 | Hainan area: 33.8 (12.1) | 55.6% |
| Su 2019 [53] | | Mainland China | Cohort | Retrospective | Patients undergoing kidney biopsy | 2007-2016 | 2,725 | 41.24 (15.18) | 55.0% |
| Tang 2017 [54] | | Mainland China | Cross-sectional | Retrospective | Primary glomerular diseases | 2010-2015 | 4,855 | 39.78 (15.43) | 53.8% |
| Tian 2020 [55] | | Mainland China | Cohort | Retrospective | Primary IgAN (age >18) | 2002-2018 | 491 | 33.08 | 52.5% |
| Wang 2018 [56] | | Mainland China | Cross-sectional | Retrospective | Children (0-18 years) undergoing kidney biopsy | 2001-2017 | 744 | NA | 37.5% |
| Wen 2021 [57] | | Mainland China | Cohort | Retrospective | Primary IgAN (Adult) | 2006-2016 | 1,096 | Male: 32.67 (11.13);  Female: 34.65 (10.76) | 56.7% |
| Wu 2020 [58] | | Mainland China | Cohort | Retrospective | Children with IgAN | 2000-2017 | 1,243 | 14 (4) | 68.0% |
| Xiao 2021 [59] | | Mainland China | Cohort | Prospective | Primary nephrotic syndrome patients | 2014-2019 | 113 | 26.8 (7.2) | 97.3% |
| Xu 2021 [60] | | Mainland China | Cohort | Retrospective | Patients who underwent renal biopsy | 2008-2019 | 3,554 | 37.5 (13.0) | 52.9% |
| Yang, Xie 2021 [61] | | Mainland China | Cohort | Retrospective | Renal biopsy cases (pediatric) | 1994-2019 | 1,459 | 10.34 (range: 6-14) | 61.1% |
| Yang, Zhang 2021 [62] | | Mainland China | Cohort | Retrospective | Inpatients with renal diseases | 2016-2019 | 252 | 40.0 (16.0) | 60.7% |
| Zhang 2021 [63] | | Mainland China | Cross-sectional | Retrospective | Children who underwent physical examination | 2017-2020 | 3,623 | 4.5 (1.11) | 51.6% |
| Zhao 2021 [64] | | Mainland China | Cohort | Retrospective | Pediatric patients with biopsy-proven IgA nephropathy and nephrotic-range proteinuria | 2011-2017 | 90 | 8.4 (3.2) | 71.1% |
| Zheng 2018 [65] | | Mainland China | Cross-Sectional Survey | Retrospective | IgAN patients | 2012-2017 | 1,350 | NA | NA |
| Zhou 2017 [66] | | Mainland China | Cross-sectional | Retrospective | primary IgAN | 2013-2014 | 375 | 36 (range: 28.0-43.0) | 51.7% |
| Zhou 2018 [67] | | Mainland China | Cross-sectional | Retrospective | Patients >15 years undergoing kidney biopsy, without renal transplantation) | 2001-2015 | 10,779 | 40 (14.86) | 51% |
| Zhu 2016 [68] | | Mainland China | Cohort | Retrospective | Patients undergoing kidney biopsy | 2009-2014 | 224 | 36.85 (14.48) | 44.6% |
| Zhu 2021 [69] | | Mainland China | Cohort | Retrospective | Biopsy-confirmed IgAN patients after renal transplantation | 2004-2017 | 102 | 35.5 (9.1) | 70.6% |

Note: * This study is 1 annual report and the prevalence data was reported. **Subgroups were listed in separate rows. ***All ages were mean values plus standard deviation unless otherwise reported.

Abbreviations: ARF, Acute renal failure; CRF, Chronic renal failure; IgAN, IgA nephropathy; IMN, Idiopathic membranous nephropathy ; NSPN, non-nephrotic syndromes proteinuria; PGN, Primary glomerulonephritis; TSN, Taiwan Society of Nephrology; WGNSSDTCRD, Working Group for National Survey on Status of Diagnosis and Treatment of Childhood Renal Diseases

Table S3. Quality Assessment of Included Articles

| First Author’ s Name (Last name, first name) | Year | Journal | What is the study design of this study? | Was the study a prospective study or a retrospective study? | In case of a case-control study, were the groups similar at the outset of the study in terms of prognostic factors? | Was the intervention used appropriately? | Were the outcome measures in the study reliable? | Were the outcome measures in the study valid? | Was the statistical analysis conducted appropriately in the study? | Was the quality of reporting appropriate in the study? | Can the study results be generalized to routine practice? |
| --- | --- | --- | --- | --- | --- | --- | --- | --- | --- | --- | --- |
| Bae, Hong Jin [22] | 2013 | Korean Journal of Medicine | Cohort study | retrospective | NA | Not clear | Not clear | Not clear | Not clear | Not clear | Not clear |
| Briganti, Esther M [1] | 2001 | Nephrol Dial Transplant | Cohort study | retrospective | NA | NA | Yes | Yes | Yes | Yes | Yes |
| Cai, Qingqing [32] | 2019 | American journal of kidney diseases | Cohort study | retrospective | NA | NA | Yes | Yes | Yes | Yes | Not clear |
| Cen, Ji [33] | 2021 | Journal of Chengdu Medical College | Cohort study | retrospective | NA | NA | Yes | Yes | Yes | Yes | Not clear |
| Chen, Liangmei [35] | 2019 | Renal failure | Case-series | retrospective | NA | NA | Yes | Yes | Yes | Yes | Not clear |
| Chen, Shasha [34] | 2016 | American journal of kidney diseases | Cohort study | retrospective | NA | NA | Yes | Yes | Yes | Yes | Not clear |
| Chiu, Hsien-Fu [4] | 2018 | BMC (BioMed Central) nephrology | Cohort study | retrospective | NA | NA | Yes | Yes | Yes | Yes | Yes |
| Cho, Byoung-Soo [23] | 2013 | Clinical and Experimental Nephrology | Cohort study | retrospective | NA | NA | No | Yes | Yes | Yes | Yes |
| Duan, Yanya [36] | 2021 | Chinese Journal of Nephrology | Cohort study | retrospective | NA | NA | Yes | Yes | Yes | NA | Not clear |
| Feng, Shipin [37] | 2021 | Journal of Clinical Pediatrics | Cohort study | retrospective | NA | NA | Yes | Yes | Yes | Yes | No |
| Goto, Masashi [7] | 2009 | Nephrol Dial Transplant | Cohort study | prospective | NA | NA | Yes | Yes | Yes | Yes | Yes |
| Gu, Cuizhi [38] | 2021 | Journal of Central South University (Medical Science) | Cohort study | retrospective | NA | NA | Yes | Yes | Yes | Yes | Not clear |
| Hattori, Motoshi [8] | 2016 | Clinical and Experimental Nephrology | Cohort study | retrospective | NA | NA | No | Yes | No | Yes | Not clear |
| Huang Liping [39] | 2019 | Journal of General Practice Dentistry (electronic version) | Cohort study | retrospective | NA | NA | Yes | Not clear | Yes | Not clear | Yes |
| Jegatheesan, Dev [2] | 2016 | Nephrology (Carlton) | Cohort study | retrospective | NA | NA | Yes | Yes | Yes | Yes | Yes |
| Jeong, Eu Gene [24] | 2017 | Saudi Journal of Kidney Diseases and Transplantation | Cohort study | retrospective | NA | Yes | Yes | Yes | Yes | Yes | Yes |
| Kaihan, Ahmad Baseer [9] | 2017 | Clinical and Experimental Nephrology | Cohort study | retrospective | NA | NA | Yes | Yes | Yes | Yes | Yes |
| Kajiwara, Nobuyuki [10] | 2020 | Clinical and Experimental Nephrology | Cohort study | prospective/retrospective | NA | NA | No | Yes | Not clear | Yes | Yes |
| Kee, Youn Kyung [25] | 2017 | Medicine (Baltimore) | Cohort study | retrospective | NA | NA | Yes | Yes | Yes | Yes | Yes |
| Komatsu, Hiroyuki [11] | 2013 | Kidney Blood Press Res | Cohort study | retrospective | NA | NA | Yes | Yes | Yes | Yes | Yes |
| Le, Weibo [40] | 2011 | Journal of Nephrology and Dialysis Renal Transplantation | Case-series | retrospective | NA | NA | Yes | Yes | Yes | Yes | Not clear |
| Lee, Adrian Y. S. [3] | 2021 | Journal of Nephropathology | Cohort study | retrospective | NA | NA | Yes | Yes | Yes | Yes | Yes |
| Lee, Ha Jung [27] | 2012 | Graduate School of Seoul National University | Cohort study | retrospective | NA | NA | Not clear | Yes | Yes | Yes | Yes |
| Lee, Hajeong [27] | 2012 | Public Library of Science One | Cohort study | retrospective | NA | NA | Yes | Yes | Yes | Yes | Yes |
| Lee, Hajeong [29] | 2013 | American journal of nephrology | Cohort study | retrospective | NA | Yes | Yes | Yes | Yes | Yes | Yes |
| Lee, Sulra [26] | 2010 | Korean J Nephrol | Cohort study | retrospective | NA | Not clear | Not clear | Not clear | Not clear | Not clear | Not clear |
| Li, Jinan [41] | 2018 | Nephrol Dial Transplant | Cohort study | retrospective | NA | NA | Yes | Yes | Yes | Yes | Yes |
| Liu, Yang [42] | 2021 | Medicina clinica | Cohort study | retrospective | NA | NA | Yes | Yes | Yes | Yes | Not clear |
| Lu, Honghua [43] | 2017 | Contemporary Medicine | Cohort study | retrospective | NA | NA | Not clear | Not clear | Yes | Not clear | Not clear |
| Lu, Xinling [44] | 2021 | Essential Health Readings | Cohort study | prospective | NA | NA | Not clear | Not clear | Yes | Not clear | Yes |
| Matsuzak, Keiichi [12] | 2013 | Clinical and Experimental Nephrology | Cohort study | prospective/retrospective | NA | NA | No | Yes | Not clear | Yes | Not clear |
| Miyabe, Yoei [13] | 2021 | Scientific Reports | Cohort study | retrospective | NA | NA | Yes | Yes | Yes | Yes | Yes |
| Moriyama, Takahito [14] | 2014 | Public Library of Science One | Cohort study | retrospective | NA | Yes | Yes | Yes | Yes | Yes | Yes |
| Nie, Ping [47] | 2019 | BioMed research international | Case-series | retrospective | NA | NA | Yes | Yes | Yes | Yes | Not clear |
| Nie, Ping [48] | 2021 | Renal failure | Cohort study | retrospective | NA | NA | Yes | Yes | Yes | Yes | Not clear |
| Nie, Sheng [46] | 2018 | Clinical journal of the American Society of Nephrology | Cohort study | prospective/retrospective | NA | NA | Yes | Yes | Yes | Yes | Yes |
| Oshima, Yasuko [15] | 2015 | International Urology and Nephrology | Cohort study | retrospective | NA | NA | Yes | Yes | Yes | Yes | Yes |
| Pan, Qingdeng [49] | 2021 | Anhui Medical and Pharmaceutical Journal | Cohort study | prospective | NA | NA | Yes | Yes | Yes | Not clear | Not clear |
| Peng, Qingquan [50] | 2015 | Medicine and Society | Case-control | retrospective | Not clear | NA | Yes | Yes | Yes | Yes | Not clear |
| Qi, Shaoqin [51] | 2021 | Diet and Health Care | Cohort study | retrospective | NA | NA | Not clear | Not clear | Yes | No | Yes |
| Sato, Ryuta [16] | 2015 | Clinical and Experimental Nephrology | Cohort study | prospective/retrospective | NA | NA | Yes | Yes | Yes | Yes | Yes |
| Shang, Ruihua [52] | 2021 | Journal of Clinical Nephrology,2021,21(2) | Cohort study | retrospective | NA | NA | Yes | Yes | Yes | Yes | No |
| Shin, Ho Sik [30] | 2017 | Renal failure | Cohort study | retrospective | NA | NA | Yes | Yes | Yes | Yes | Yes |
| Su, Sensen [53] | 2019 | Medicine (Baltimore) | Cohort study | retrospective | NA | NA | Yes | Yes | Yes | Yes | Not clear |
| Sugiyama, Hitoshi [17] | 2013 | Clinical and Experimental Nephrology | Case-series | prospective | NA | NA | Yes | Yes | Yes | Yes | Not clear |
| Suh, Jin-Soon [31] | 2020 | Journal of Clinical Medicine | Cohort study | retrospective | NA | NA | Yes | Yes | Yes | Yes | Yes |
| Tang, Lijun [54] | 2017 | Nephrology (Carlton) | Case-series | retrospective | NA | NA | Yes | Yes | Yes | Yes | Not clear |
| Tian, Shasha [55] | 2020 | International immunopharmacology | Cohort study | retrospective | NA | NA | Yes | Yes | Yes | Not clear | Not clear |
| Utsunomiya, Yasushi [18] | 2003 | Pediatric Nephrol | Cohort study | prospective/retrospective | NA | NA | No | Yes | Yes | Yes | Yes |
| Wang, Nini [56] | 2018 | The Journal of international medical research | Case-series | retrospective | NA | NA | Yes | No | Yes | Yes | Not clear |
| Wen, Dongmei [57] | 2021 | International Urology and Nephrology | Cohort study | retrospective | NA | NA | Yes | Yes | Yes | Yes | Not clear |
| WGNSSDTCRD [45] | 2013 | Zhonghua er ke za zhi | Case-series | retrospective | NA | NA | Yes | Yes | Yes | Yes | Yes |
| Wu, Heyan [58] | 2020 | BMC (BioMed Central) nephrology | Cohort study | retrospective | NA | NA | Yes | Yes | Yes | Yes | Not clear |
| Xiao, Liaoyuan [59] | 2021 | Northwest Journal of Defense Medicine | Cohort study | prospective | NA | NA | Yes | Yes | Yes | Yes | No |
| Xu, Ziwu [60] | 2021 | Anhui Medical University (Thesis) | Cohort study | retrospective | NA | NA | Yes | Yes | Yes | Not clear | Not clear |
| Yamamoto, Ryohei | 2010 | American journal of kidney diseases | Cohort study | prospective | NA | Yes | Yes | Yes | Yes | Yes | Yes |
| Yang, Dan [61] | 2021 | Chinese Healing Medicine | Cohort study | retrospective | NA | NA | Yes | Yes | Yes | NA | Not clear |
| Yang, Jing [62] | 2021 | Tibetan Medicine | Cohort study | retrospective | NA | NA | Yes | Yes | Yes | Yes | Not clear |
| Yata, Nahoko [20] | 2008 | Pediatric Nephrol | Cohort study | retrospective | NA | NA | Yes | Yes | Yes | Yes | Yes |
| Yokoyama, Hitoshi [21] | 2012 | Clinical and Experimental Nephrology | Cohort study | prospective/retrospective | NA | NA | Yes | Yes | Yes | Yes | Yes |
| Yu, Mei-Ching [6] | 2014 | Biomedical journal | Case-series | retrospective | NA | NA | Yes | Yes | Yes | Yes | Yes |
| Zhang, Pengcheng [63] | 2021 | World's Newest Medical Information Digest | Case-series | retrospective | NA | NA | Yes | No | Yes | Yes | No |
| Zhao, Jingli [64] | 2021 | Medicine (Baltimore) | Cohort study | retrospective | NA | NA | Yes | Yes | Yes | Yes | Yes |
| Zheng, Xiao [65] | 2018 | Chinese Journal of Evidence-Based Medicine | Case-series | retrospective | NA | NA | Yes | NA | Yes | NA | Not clear |
| Zhou, Qin [67] | 2018 | Renal failure | Cohort study | retrospective | NA | NA | Yes | Yes | Yes | Yes | Yes |
| Zhou, Shanshan [66] | 2017 | Clinical Nephrology | Case-series | retrospective | NA | NA | Yes | Not clear | Yes | Yes | Not clear |
| Zhu, Linqiao [69] | 2021 | General Practice Nursing | Cohort study | retrospective | NA | NA | Yes | Not clear | Yes | Yes | No |
| Zhu, Zaizhi [68] | 2016 | Huaxi Medicine | Cohort study | retrospective | NA | NA | Yes | Yes | Yes | Yes | No |

Abbreviations: NA, not applicable; WGNSSDTCRD, Working Group for National Survey on Status of Diagnosis and Treatment of Childhood Renal Diseases

* The intervention was a clinical nursing pathway.

Figure S1. Hospitalization cost per capita per year for IgAN patients in mainland China

# References

1. Briganti EM, Dowling J, Finlay M, Hill PA, Jones CL, Kincaid-Smith PS, et al. The incidence of biopsy-proven glomerulonephritis in Australia. Nephrology, Dialysis, Transplantation: Official Publication of the European Dialysis and Transplant Association - European Renal Association. 2001;16(7):1364-7.

2. Jegatheesan D, Nath K, Reyaldeen R, Sivasuthan G, John GT, Francis L, et al. Epidemiology of biopsy-proven glomerulonephritis in Queensland adults. Nephrology (Carlton). 2016;21(1):28-34.

3. Lee AYS, Lin M-W. Do IgA nephropathy presentations display any seasonality? Journal of Nephropathology. 2021;10(3):e33-e.

4. Chiu HF, Chen HC, Lu KC, Shu KH, Taiwan Society of N. Distribution of glomerular diseases in Taiwan: preliminary report of National Renal Biopsy Registry-publication on behalf of Taiwan Society of Nephrology. BMC Nephrology. 2018;19(1):6.

5. National Health Research Institute & Taiwan Society of Nephrology. 2019 Annual Report on Kidney Disease in Taiwan.

6. Yu MC, Lee F, Huang WH, Hsueh S. Percutaneous ultrasound-guided renal biopsy in children: the need for renal biopsy in pediatric patients with persistent asymptomatic microscopic hematuria. Biomedical Journal. 2014;37(6):391-7.

7. Goto M, Wakai K, Kawamura T, Ando M, Endoh M, Tomino Y. A scoring system to predict renal outcome in IgA nephropathy: a nationwide 10-year prospective cohort study. Nephrology, Dialysis, Transplantation: Official Publication of the European Dialysis and Transplant Association - European Renal Association. 2009;24(10):3068-74.

8. Hattori M, Iwano M, Sako M, Honda M, Okada H, Akioka Y, et al. Transition of adolescent and young adult patients with childhood-onset chronic kidney disease from pediatric to adult renal services: a nationwide survey in Japan. Clinical and Experimental Nephrology. 2016;20(6):918-25.

9. Kaihan AB, Yasuda Y, Katsuno T, Kato S, Imaizumi T, Ozeki T, et al. The Japanese Histologic Classification and T-score in the Oxford Classification system could predict renal outcome in Japanese IgA nephropathy patients. Clinical and Experimental Nephrology. 2017;21(6):986-94.

10. Kajiwara N, Hayashi K, Fujiwara M, Nakayama H, Ozaki Y. Identification of children with chronic kidney disease through school urinary screening using urinary protein/creatinine ratio measurement: an observational study. Clinical and Experimental Nephrology. 2020;24(5):450-7.

11. Komatsu H, Kikuchi M, Nakagawa H, Fukuda A, Iwakiri T, Toida T, et al. Long-term survival of patients with IgA nephropathy after dialysis therapy. Kidney and Blood Pressure Research. 2013;37(6):649-56.

12. Matsuzaki K, Suzuki Y, Nakata J, Sakamoto N, Horikoshi S, Kawamura T, et al. Nationwide survey on current treatments for IgA nephropathy in Japan. Clinical and Experimental Nephrology. 2013;17(6):827-33.

13. Miyabe Y, Karasawa K, Akiyama K, Ogura S, Takabe T, Sugiura N, et al. Grading system utilising the total score of Oxford classification for predicting renal prognosis in IgA nephropathy. Scientific Reports. 2021;11(1):3584.

14. Moriyama T, Tanaka K, Iwasaki C, Oshima Y, Ochi A, Kataoka H, et al. Prognosis in IgA nephropathy: 30-year analysis of 1,012 patients at a single center in Japan. PLoS ONE. 2014;9(3):e91756.

15. Oshima Y, Moriyama T, Itabashi M, Takei T, Nitta K. Characteristics of IgA nephropathy in advanced-age patients. International Urology and Nephrology. 2015;47(1):137-45.

16. Sato R, Joh K, Komatsuda A, Ohtani H, Okuyama S, Togashi M, et al. Validation of the Japanese histologic classification 2013 of immunoglobulin A nephropathy for prediction of long-term prognosis in a Japanese single-center cohort. Clinical and Experimental Nephrology. 2015;19(3):411-8.

17. Sugiyama H, Yokoyama H, Sato H, Saito T, Kohda Y, Nishi S, et al. Japan Renal Biopsy Registry and Japan Kidney Disease Registry: Committee Report for 2009 and 2010. Clinical and Experimental Nephrology. 2013;17(2):155-73.

18. Utsunomiya Y, Koda T, Kado T, Okada S, Hayashi A, Kanzaki S, et al. Incidence of pediatric IgA nephropathy. Pediatric Nephrology. 2003;18(6):511-5.

19. Yamamoto R, Nagasawa Y, Shoji T, Iwatani H, Hamano T, Kawada N, et al. Cigarette smoking and progression of IgA nephropathy. Am J Kidney Dis. 2010;56(2):313-24.

20. Yata N, Nakanishi K, Shima Y, Togawa H, Obana M, Sako M, et al. Improved renal survival in Japanese children with IgA nephropathy. Pediatric Nephrology. 2008;23(6):905-12.

21. Yokoyama H, Sugiyama H, Sato H, Taguchi T, Nagata M, Matsuo S, et al. Renal disease in the elderly and the very elderly Japanese: analysis of the Japan Renal Biopsy Registry (J-RBR). Clinical and Experimental Nephrology. 2012;16(6):903-20.

22. Bae HJ, Moon KR, Kim YJ, Choi DE, Na KR, Lee KW, et al. Clinical and histopathological analysis of the kidney biopsies of 2,450 patients seen over 30 years at Chungnam National University Hospital. Korean Journal of Medicine. 2015;84(3):379-88.

23. Cho BS, Hahn WH, Cheong HI, Lim I, Ko CW, Kim SY, et al. A nationwide study of mass urine screening tests on Korean school children and implications for chronic kidney disease management. Clinical and Experimental Nephrology. 2013;17(2):205-10.

24. Jeong EG, Hyoun S, Lee SM, An WS, Kim SE, Son YK. Clinical outcomes of nephrotic syndrome in immunoglobulin a nephropathy. Saudi Journal of Kidney Diseases and Transplantation. 2017;28(6):1314-20.

25. Kee YK, Yoon CY, Kim SJ, Moon SJ, Kim CH, Park JT, et al. Determination of the optimal target level of proteinuria in the management of patients with glomerular diseases by using different definitions of proteinuria. Medicine (Baltimore). 2017;96(44):e8154.

26. Lee S, Choi S, Se-bin S, Kyunghwan J, Taewon L. Relative risk factors of prognosis in IgA nephropathy patients with depressed renal functions. Korean J Nephrol. 2010;29(2):198-207.

27. Lee H, Kim DK, Oh KH, Joo KW, Kim YS, Chae DW, et al. Mortality of IgA nephropathy patients: a single center experience over 30 years. PLoS ONE. 2012;7(12):e51225.

28. Ha-jeong L. Long-term patient and renal survivals and their predictable factor analyses in IgA nephropathy patients [Thesis]: [Seoul National University Graduate School]; 2012.

29. Lee H, Kim DK, Oh KH, Joo KW, Kim YS, Chae DW, et al. Mortality and renal outcome of primary glomerulonephritis in Korea: observation in 1,943 biopsied cases. American Journal of Nephrology. 2013;37(1):74-83.

30. Shin HS, Cho DH, Kang SK, Kim HJ, Kim SY, Yang JW, et al. Patterns of renal disease in South Korea: a 20-year review of a single-center renal biopsy database. Ren Fail. 2017;39(1):540-6.

31. Suh JS, Jang KM, Hyun H, Cho MH, Lee JH, Park YS, et al. Remission of proteinuria may protect against progression to chronic kidney disease in pediatric-onset IgA nephropathy. J Clin Med. 2020;9(7):2058.

32. Cai Q, Shi S, Wang S, Ren Y, Hou W, Liu L, et al. Microangiopathic lesions in IgA nephropathy: a cohort study. Am J Kidney Dis. 2019;74(5):629-39.

33. Cen J, Hu H, Cheng Y, Liu Y, Wu S, Qin W, et al. Guangxi duominzu juju didu dan zhongxin shenhuojian bingli ziliao ji minzu tedian fenxi [Pathological data of single-center kidney biopsy and analysis of ethnic characteristics in a multi-ethnic area of Guangxi]. Journal of Chengdu Medical College. 2021;16(04):482-5+511.

34. Chen S, Tang Z, Xiang H, Li X, Chen H, Zhang H, et al. Etiology and outcome of crescentic glomerulonephritis from a single center in china: a 10-year review. Am J Kidney Dis. 2016;67(3):376-83.

35. Chen L, Luodelete M, Dong C, Li B, Zhang W, Nie P, et al. Pathological spectrum of glomerular disease in patients with renal insufficiency: a single-center study in Northeastern China. Ren Fail. 2019;41(1):473-80.

36. Duan Y, Lie C, Zhang L, AYiJiaKen K, Guo W, Li Y, et al. Xinjiang weiwuer zizhiqu 10 684 li shenhuojian bingli ziliao yu liuxingbingxue tedian fenxi [Analysis of pathological data and epidemiological characteristics of 10 684 kidney biopsies in Xinjiang Uygur Autonomous Region]. Chinese Journal of Nephrology. 2021;37(06):490-8.

37. Feng S, Wang L, Liu X, Luo W, Xie M, Yang Q. 1002 li manxing shenzangbing huaner linchuang ji bingli fenxi [Clinical and pathological analysis of 1002 children with chronic kidney disease]. Journal of Clinical Pediatrics. 2021;39(02):87-90.

38. Gu C, Li Q, Liang W, Bi H, Xie M, Wu D. Guilin he jining liangsuo yiyuan 1370 li shenhuojian jibing fenbu tezheng [Characteristics of disease distribution in 1370 kidney biopsies from two hospitals in Guilin and Jining]. Journal of Central South University (Medical Edition). 2021;46(09):974-82.

39. Huang L. Tanjiu dui huanyou butong dengji IgA shenbing de huanzhe yuyi gexinghua huli ganyu duiyu xinli qingxu yiji shenghuo zhiliang de yingxiang [Investigation of the effect of personalized nursing interventions on psychological, emotional and quality of life in patients with different grades of IgA nephropathy (IgAN)]. Journal of General Practice Dentistry (electronic version). 2019;6(25):110-4.

40. Le W, Liang S, Deng K, Hu Y, Zeng C, Liu D. 1126 li zhongguo hanzu chengren IgA shenbing huanzhe de changqi yuhou ji weixian yinsu fenxi [Long-term prognosis and risk factor analysis of 1126 Chinese Han adult patients with IgA nephropathy]. Journal of Nephrology and Dialysis Renal Transplantation. 2011;20(02):101-8.

41. Li J, Cui Z, Long J, Huang W, Wang J, Zhang H, et al. Primary glomerular nephropathy among hospitalized patients in a national database in China. Nephrology, Dialysis, Transplantation: Official Publication of the European Dialysis and Transplant Association - European Renal Association. 2018;33(12):2173-81.

42. Liu Y, Wei W, Yu C, Xing L, Wang M, Liu R, et al. Epidemiology and risk factors for progression in Chinese patients with IgA nephropathy. Med Clin (Barc). 2021;157(6):267-73.

43. Lu H, Xiao L, Lu X, Liang J. Gexinghua huli moshi dui IgA shenbing huanzhe qingxu ji shenghuo zhiliang yingxiang de yanjiu [The effect of personalized nursing mode on the emotion and quality of life of patients with IgA nephropathy]. Contemporary Medicine. 2017;23(5):30-3.

44. Lu X. Zhendui butong fenji IgA shenbing huanzhe kaizhan gexinghua huli moshi de linchuang xiaoguo guancha [Clinical effects of personalized care model for patients with different grades of IgA nephropathy]. Essential Health Readings. 2021(8):125.

45. Working Group for National Survey on Status Diagnosis and Treatment of Childhood Renal Diseases. [Multicenter investigation of therapeutic status of children with IgA nephropathy in China]. Zhonghua Er Ke Za Zhi. 2013;51(7):486-90.

46. Nie S, He W, Huang T, Liu D, Wang G, Geng J, et al. The spectrum of biopsy-proven glomerular diseases among children in China: a national, cross-sectional survey. Clin J Am Soc Nephrol. 2018;13(7):1047-54.

47. Nie P, Chen R, Luo M, Dong C, Chen L, Liu J, et al. Clinical and pathological analysis of 4910 patients who received renal biopsies at a single center in Northeast China. Biomed Res Int. 26 Mar 2019;2019:6869179.

48. Nie P, Lou Y, Wang Y, Bai X, Zhang L, Jiang S, et al. Clinical and pathological analysis of renal biopsies of elderly patients in Northeast China: a single-center study. Ren Fail. 2021;43(1):851-9.

49. Pan Q, Ye Z, Zeng C, Ning W. Feishenxing tefaxing moxing shenbing yu feishenxing IgA shenbing de linchuang tedian bijiao [Clinical comparative analysis of non-nephrotic idiopathic membranous nephropathy and non-nephrotic IgA nephropathy]. Anhui Medicine. 2021;25(2):268-70.

50. Peng Q, Xu G, Zhang C, Fang P. Wuhanshi mou sanjia yiyuan IgA shenbing shenchuanci huojian huanzhe linchuang lujing shishi xiaoguo pingjia [Evaluation of clinical pathway implementation effect in patients with IgA nephropathy renal puncture biopsy in a tertiary hospital in Wuhan]. Medicine and Society. 2015;28(10):18-20.

51. Qi S. Zhendui butong fenji IgA shenbing huanzhe kaizhan gexinghua huli moshi de linchuang xiaoguo guancha [Clinical effects of personalized care model for patients with different grades of IgA nephropathy]. Diet and Health Care. 2021;8.

52. Shang R, Zhu Y, Lin Z, Ma D, Ma Y, Ji M, et al. Yu qiong liangdi yuanfaxing shenxiaoqiu jibing bingli leixing de bianqian duibi ji linchuang fenxi [Comparison and clinical analysis of pathological types of primary glomerular diseases in North Henan and Hainan]. J Clin Nephrol. 2021;21(2):111-8.

53. Su S, Yu J, Wang Y, Wang Y, Li J, Xu Z. Clinicopathologic correlations of renal biopsy findings from northeast China: A 10-year retrospective study. Medicine (Baltimore). 2019;98(23):e15880.

54. Tang L, Yao J, Kong X, Sun Q, Wang Z, Zhang Y, et al. Increasing prevalence of membranous nephropathy in patients with primary glomerular diseases: a cross-sectional study in China. Nephrology (Carlton). 2017;22(2):168-73.

55. Tian S, Yang X, Luo J, Guo H. Clinical and prognostic significance of C1q deposition in IgAN patients-a retrospective study. Int Immunopharmacol. 2020;88:106896.

56. Wang N, Zhu T, Tao Y. Clinicopathological features of pediatric renal biopsies in the plateau regions of China. Journal of International Medical Research. 2018;46(11):4539-46.

57. Wen D, Tang Y, Tan L, Tan J, Chen D, Zhang Y, et al. Sex disparities in IgA nephropathy: a retrospective study in Chinese patients. International Urology and Nephrology. 2021;53(2):315-23.

58. Wu H, Xia Z, Gao C, Zhang P, Yang X, Wang R, et al. The correlation analysis between the Oxford classification of Chinese IgA nephropathy children and renal outcome – a retrospective cohort study. BMC Nephrology. 2020;21(1):247.

59. Xiao L, Wang J, Zhang M, He X, Gao J, Xi C. Yufangxing kangning zai budui guanbing shenbing zonghezheng zhiliao zhong de yingyong xiaoguo yanjiu [Study on the effect of preventive anticoagulation in the treatment of nephrotic syndrome in army officers and soldiers]. Northwest Journal of Defense Medicine. 2021;42(01):30-6.

60. Xu Z, Xiong Z. 3554 li shenzang bingli yu linchuang xiangguanxing fenxi [Analysis of renal pathology and clinical correlation in 3554 cases] [Master, 10.26921/d.cnki.ganyu.2021.001127]: M.S., Anhui Medical University; 2021.

61. Yang D, Xie Y, He Z, Li Y, Li C. Qinhuangdao shi 1459 li xueling ertong shenzang jibing linchuang yu bingli fenxi [Clinical and pathological analysis of 1459 cases of renal disease in school-age children in Qinhuangdao]. Chinese Healing Medicine. 2021;30(06):640-3.

62. Yang J, Zhang L, Wang Y. Manxing shenzangbing shen chuanci huojian bingli tezheng fenxi [Analysis of pathological features of renal puncture biopsy in chronic kidney disease]. Tibetan Medicine. 2021;42(05):49-51.

63. Zhang P, Chen Z, Liu M. Huizhou shi dayawan diqu xuelingqian ertong niaoye shaicha fenxi [Analysis of urine screening in preschool children in Dayawan, Huizhou]. World's Newest Medical Information Digest. 2021;21(84).

64. Zhao JL, Wang JJ, Huang GP, Feng CY. Primary IgA nephropathy with nephrotic-range proteinuria in Chinese children. Medicine (Baltimore). 2021;100(21):e26050.

65. Zheng X, Zhang J, Lu C. Xinjiang weiwuer zizhiqu renmin yiyuan 2012~2017 nian manxing shenzangbing huanzhe de jibing goucheng ji yiliao feiyong de hengduanmian diaocha [Disease composition and medical expenses of chronic kidney disease in People's Hospital of Xinjiang Uygur Autonomous Region from 2012 to 2017: a cross-sectional survey]. Chin J Evid-Based Med. 2018;18(9):903-6.

66. Zhou S, Fu J, Liu M, Yang S, Zhou Q, Yu X, et al. The prevalence and risk factors of abnormal circadian blood pressure in patients with IgA nephropathy. Clinical Nephrology. 2017;88(12):344-53.

67. Zhou Q, Yang X, Wang M, Wang H, Zhao J, Bi Y, et al. Changes in the diagnosis of glomerular diseases in east China: a 15-year renal biopsy study. Ren Fail. 2018;40(1):657-64.

68. Zhu Z, Zou Q, Chen Y, Hu F, Bai J, Chao Q, et al. 224 li shen huoti zuzhi jiancha de linchuang lujing yu bingli fenxi [Analysis of the clinical pathway and pathologic features of 224 cases of renal biopsy]. Huaxi Medicine. 2016;31(5):845-9.

69. Zhu L, Huang X, Zhang J, Li W, Chen E, Guo N. 102 li yizhishen IgA shenbing de huli tihui [Nursing experience of 102 cases of IgA nephropathy in transplanted kidneys]. General Practice Nursing. 2021;19(04):513-5.
